# Growth dynamics and compensatory mechanisms in fish under fasting and refeeding regimes

**DOI:** 10.3389/fphys.2026.1799460

**Published:** 2026-06-01

**Authors:** Mohsen A. Khormi

**Affiliations:** Department of Biology, College of Science, Jazan University, Jazan, Saudi Arabia

**Keywords:** aquaculture, compensatory growth, fasting, feed management, fish physiology, refeeding

## Abstract

Fasting and refeeding strategies have gained prominence in aquaculture as management tools that may influence growth performance, feed utilization, and physiological status in cultured fish. This review synthesizes current research on the effects of controlled fasting and subsequent refeeding across multiple species, highlighting their impact on growth responses, and metabolic regulation. During fasting, many fish species exhibit reduced somatic growth, decreased feed utilization efficiency, and metabolic adjustments aimed at conserving energy reserves. Upon refeeding, some species demonstrate a compensatory growth (CG) response characterized by hyperphagia, accelerated weight gain, and shifts in nutrient allocation. This response has been associated, in certain species and experimental conditions, with modulation of growth-related gene expression, replenishment of hepatic energy stores, and partial recovery of biochemical indicators. The magnitude and efficiency of CG vary widely depending on fasting duration, refeeding strategy, species-specific metabolic capacity, life stage, and environmental conditions. Strategic implementation of fasting-refeeding protocols can reduce feed costs, mitigate overfeeding-related stress, and enhance sustainability in aquaculture operations. These findings support the integration of tailored fasting regimes into aquaculture management to maximize growth recovery, maintain metabolic health, and improve overall production efficiency.

## 1 Introduction

Periods of food scarcity are commonly tolerated by many fish species in their natural environment and are caused by factors such as seasonal changes, prey availability, migration and spawning (Ali et al., 2003; Binner et al., 2008; Cipriano et al., 2015). Fasting generally suppresses somatic growth, as energy allocation shifts toward maintenance metabolism and survival (Rungruangsak-Torrissen et al., 2006; Caruso et al., 2014). Successful adaptation to food deprivation depends primarily on the mobilization and regulation of energy reserves, including carbohydrates, lipid stores, and muscle proteins. These energy reserves are utilized to sustain essential physiological functions and preserve vital organ integrity until feeding is resumed (McCue, 2010; Furné et al., 2012). This metabolic transition is typically characterized by enhanced catabolic activity, reduced protein deposition, and altered endocrine signaling (Nebo et al., 2013; Otsu et al., 2025).

When feeding resumes, some fish species exhibit compensatory growth (CG), a phenomenon involving accelerated growth relative to continuously fed individuals. In fish, this response represents an adaptive physiological strategy that enables individuals to recover part or all the growth lost during fasting periods. At the organismal level, CG is typically characterized by increased feed intake (hyperphagia), enhanced feed conversion efficiency, and rapid somatic growth following refeeding (Reigh et al., 2006; Jobling, 2010; Kim et al., 2022). These responses are supported by coordinated endocrine regulation involving key growth-related hormones such as growth hormone (GH), insulin-like growth factor I (IGF-I), and insulin, which collectively regulate nutrient partitioning and tissue growth (Butler and Le Roith, 2001; Davis and Gaylord, 2011; Chen et al., 2019). At the molecular level, refeeding activates anabolic pathways and stimulates the expression of growth-related genes in metabolically active tissues, particularly liver and skeletal muscle, thereby promoting protein synthesis and tissue accretion (Hagen et al., 2009; Nebo et al., 2013; Elbialy et al., 2022). However, the magnitude and efficiency of compensatory growth vary widely among fish species and depend on several interacting factors including the duration of fasting, developmental stage, environmental conditions, and nutritional management strategies, and may result in partial, complete, or absent compensation (Yılmaz and Eroldogan, 2011; Torfi Mozanzadeh et al., 2015; Nebo et al., 2017; Kim et al., 2022).

In aquaculture, controlled fasting–refeeding regimes have been explored as management tools that may improve feed utilization efficiency, facilitate handling procedures, and support water quality management (Gibson Gaylord and Gatlin, 2001; Ali et al., 2003; Davis and Gaylord, 2011) Short fasting periods are also sometimes applied for gut evacuation prior to transport or harvesting, or for modifying body composition and fillet quality (Grigorakis and Alexis, 2005; Hvas et al., 2020). Nevertheless, fasting can also induce physiological stress responses, including oxidative imbalance and depletion of antioxidant defenses in certain tissues such as the liver (barım öz, 2018).

At the molecular level, fasting and refeeding influence gene expression patterns related to energy metabolism, protein turnover, and muscle growth (Joulia et al., 2003; Johansen and Overturf, 2006; Hagen et al., 2009; Elbialy et al., 2022). These responses are complex and species-dependent, reflecting differences in ecological strategies and metabolic plasticity (Luo et al., 2009; Takahashi et al., 2011; Trombley et al., 2012; Urbinati et al., 2014; Yengkokpam et al., 2014). Accordingly, it is useful to study the physiological changes that occur in fish under fasting conditions to formulate optimal feeding strategies. Despite the growing number of studies exploring fasting and refeeding responses in fish, the underlying molecular and physiological mechanisms that govern these adaptations remain fragmented and not comprehensively integrated across species. Moreover, there is currently a need for a unified synthesis that links these responses to practical aquaculture applications, particularly under modern production challenges. Therefore, the aim of this review is to critically evaluate the physiological responses of fish to fasting and refeeding strategies, with a focus on their implications for growth performance, metabolic recovery, and aquaculture sustainability. This review integrates physiological, metabolic, and molecular responses across multiple fish species, providing a comparative perspective that has not been comprehensively addressed in previous reviews.

## 2 Effects of fasting duration on body indices and compensatory growth

Growth performance serves as a key physiological metric for assessing the impact of fasting in fish. Indicators like length, weight gain, specific growth rate (SGR), and condition factor (K) offer valuable insights into how feed deprivation affects energy usage and triggers compensatory mechanisms. During fasting, most fish depend on their internal energy reserves, which commonly results in slowed or stagnant growth, although the magnitude of this response varies among species and life stages (Peres et al., 2011; Liu et al., 2020; Elbialy et al., 2022).

The K is a sensitive indicator of nutritional status, reflecting changes in body weight relative to length. Short fasting periods (typically lasting a few days) generally cause only slight declines in K, as energy demands are initially met through mobilization of endogenous reserves. In most teleost species, glycogen is utilized first, followed by lipid stores, whereas prolonged fasting may eventually lead to protein catabolism (Jobling, 2010). However, species-specific differences exist; for example, salmonids rely predominantly on lipid reserves during fasting and tend to spare protein for a longer duration compared with many other fish species (Bower et al., 2009; Lai et al., 2025). In some species or under specific fasting conditions, K may remain unchanged (Hvas et al., 2020; Kim et al., 2022; Porto et al., 2024). Extended feed deprivation, by contrast, results in greater reductions in K due to muscle protein catabolism that helps maintain metabolic balance (Wang et al., 2006; Porto et al., 2024). Upon refeeding, recovery of K is frequently observed, although the rate and extent of restoration differ considerably depending on fasting duration, feeding intensity, and species-specific metabolic traits (Ali et al., 2003; Cassidy et al., 2018).

Experimental evidence illustrates this variability. For example, (Messina et al., 2023) observed that a three-week period of feed restriction in rainbow trout (*Oncorhynchus mykiss*) led to a significant reduction in body weight, while K remained unaffected. This stability in K may be attributed to the initial size of the fish and the relatively short duration of feed deprivation. In some species, particularly during early growth stages, length may continue to increase despite reductions in body mass. This pattern has been interpreted as differential regulation of skeletal growth and tissue deposition, although its functional significance remains debated (Ali et al., 2003; Pascual et al., 2003; Favero et al., 2018). Reports of this response have been documented in species such as Atlantic salmon and gilthead sea bream (Reimers et al., 1993; Eroldoğan et al., 2006), but it is not consistently observed across taxa. In many cases, body mass responds more rapidly to nutritional stress than length, and during refeeding, weight recovery is typically more pronounced than length acceleration (Wang et al., 2006; Hvas et al., 2022). The distinct divergence of weight and length underscore the value of assessing both metrics concurrently, with their ratio reflected in the K offering a more comprehensive view of the fish’s health and growth performance.

**Figure 1:**
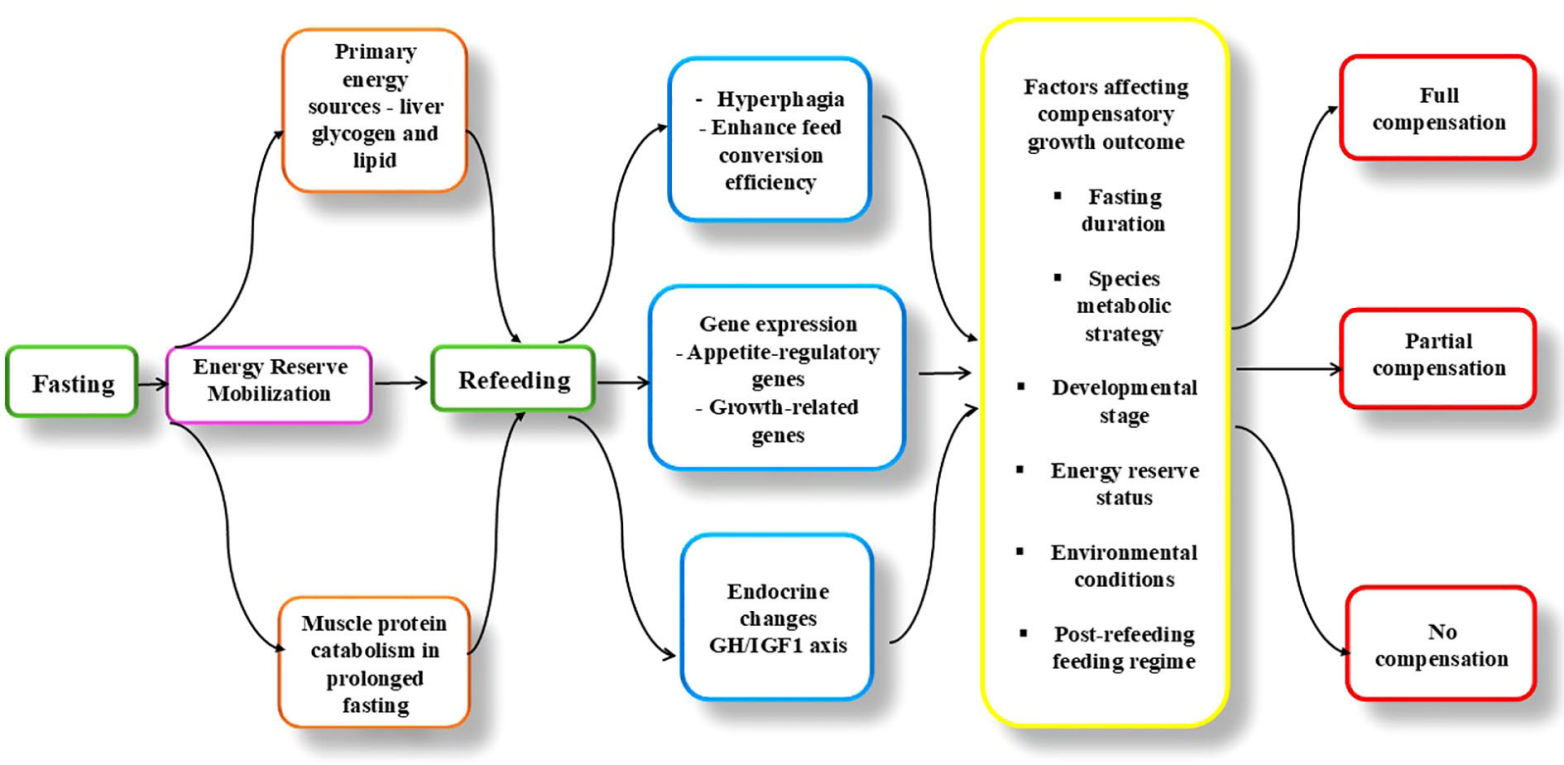
Physiological responses and molecular mechanisms of fasting and refeeding in fish.

Following refeeding, many fish species display CG, a physiological response that enables them to regain lost body mass and, in some cases, fully catch up to peers that were continuously fed (Oh et al., 2013; Simó et al., 2022). The efficiency of CG depends on hormonal regulation, shifts in metabolic efficiency, and nutrient allocation (Ali et al., 2003; Braz et al., 2022). This phenomenon is recognized as an adaptive strategy to cope with natural variations in food availability and is typically driven by mechanisms such as hyperphagia, enhanced feed conversion efficiency (FCE), shifts in energy allocation, and metabolic adjustments (Reigh et al., 2006; Jobling, 2010; Kim et al., 2022). For CG to take place effectively, certain physiological feedback mechanisms must adjust accordingly. These include shifts in the relative sizes of organs and tissues, as well as changes in their chemical composition and that of the entire body. Such adaptations help the animal meet specific growth-related targets following periods of feed deprivation or stress (Jobling, 2010). **Figure 1** illustrates the physiological responses and molecular mechanisms of fasting and refeeding in fish.

The timing of CG onset likewise varies across species. Accelerated growth responses have been reported within the first week of refeeding in species such as golden pompano (*Trachinotus ovatus*) (Liu et al., 2022), and rainbow trout (*Oncorhynchus mykiss*) (Rescan et al., 2017), grass carp (*Ctenopharyngodon idella*) (He et al., 2015), blunt snout bream (*Megalobrama amblycephala*) (Zhu et al., 2014), and Holland’s Carp *(Spinibarbus hollandi* (Yang et al., 2019), whereas in hybrid grouper (*Epinephelus fuscoguttatus*♀×*E. lanceolatus*♂), gilthead seabream (*Sparus aurata*), seabream (*Sparidentex hasta*) rainbow trout *Oncorhynchus mykiss*) recovery may be slower or incomplete (Peres et al., 2011; Torfi Mozanzadeh et al., 2015; Philip et al., 2018; Liu et al., 2020). These differences highlight the need to interpret CG within a species-specific and experimental context rather than assuming a universal response.

The effect of fasting on fish growth is closely tied to the duration of feed deprivation. **Table 1** outlines the impact of various fasting periods on growth performance and CG in various fish species. Short-term fasting is generally well tolerated by fish and has minimal influence on growth because energy demands during brief feed deprivation are met through the mobilization of glycogen and lipid reserves while protein breakdown remains low (Qosimah et al., 2025). Upon refeeding, many species exhibit rapid CG driven by increased feed intake and FCE, allowing them to exceed continuously fed groups (Hvas et al., 2020; Liu et al., 2022).

**Table 1:** Growth performance and compensatory growth in response to fasting and refeeding.

Fasting and refeeding duration	Species	Impacts of fasting	Compensatory growth outcomes	References
1-day fast, 5–6 days refeed (16 weeks)	Blackhead seabream (*Acanthopagrus schlegelii schlegelii*)	− No difference in survivability, and weight gain. − Total feed intake and daily feed intake decreased with the increase of the fasting frequency. − Actual feed intake, feed efficiency and protein efficiency ratio increased with the increase of the fasting frequency. − Proximate composition of the whole body of fish was not affected by different feeding cycles.	Complete	(Yarmohammadi et al., 2013)
2, 4 or7 days fast and refeed for 7 weeks	Gilthead seabream (*Sparus aurata*)	− Growth performance, feed intake, weight gain and feed efficiency was the highest in the control group than other fasted groups	Partial	(Eroldoğan et al., 2006)
1–2 week fast, 8 weeks refeed	Gilthead seabream (*Sparus aurata*)	− Significant loss in body weight − Intense mobilization of liver lipid and glycogen − In re-feeding period no recovery of body weight lost, and feed intake and feed efficiency were identical in all groups.	None	(Peres et al., 2011)
1-day fast, 3 and 5 days refeed (60 days)	Gilthead seabream (*Sparus aurata*)	− Decreased final weight − The highest body protein content was found in the control group	Partial	(Yılmaz and Eroldogan, 2011)
2, 4, or 8-day fasts with cyclical refeeding of 8, 16, or 32 days (80 days)	Yellowfin seabream (*Acanthopagrus latus*)	− Fish fast for 2 days and refed for 8 days showed the lowest total length. − No significant differences in growth − Fish fast for 2 days and refed for 16 days had the highest hepatosomatic indices.	Complete	(Tamadoni et al., 2020)
1–3 week fast and 5 weeks refeed (8-weeks)	Yellowfin seabream (*Acanthopagrus latus*)	− Reduced body weight; mobilization of liver and visceral energy reserves	Partial	(Torfi Mozanzadeh et al., 2021)
1, 2, 3 or 6-day fast, equal refeed (60 days)	Sobaity seabream (*Sparidentex hasta*)	− As the length of fasting increased, the compensation coefficients in feed intake and weight gain decreased.	None	(Torfi Mozanzadeh et al., 2015)
1–3 week fast and 5 weeks refeed (8-weeks)	Sobaity seabream (*Sparidentex hasta*)	− Decreased survival rate − Reduced body weight; mobilization of liver and visceral energy reserves	Complete	(Torfi Mozanzadeh et al., 2021)
118-day fast	Rainbow trout (*Oncorhynchus mykiss*)	− Declines in final length, weight, weight gain, and SGR	None	(Philip et al., 2018)
3 weeks fasting and 2 weeks refeeding	Rainbow trout (*Oncorhynchus mykiss*)	− Fasting reduced significantly the body weight (by 44%), VSI and HSI − After refeeding body weight was significantly decreased by 18% than the control and the VSI and HIS were significantly increased. The K value was unresponsive.	Partial	(Messina et al., 2023)
30 days fast and refed for 36 days	Rainbow trout (*Oncorhynchus mykiss*)	− Decrease in total body weight and K after fasting that was followed by an increase in total body weight after refeeding − Upregulation of genes promoting myofibre growth after refeeding	Complete	(Rescan et al., 2017)
10,15 days fasting and refeed 30, 25 days respectively	Nile tilapia (*Oreochromis niloticus*)	− 15 days fast decrease final weight, feed conversion, protein efficiency ratio, and survival. While, 10-day fast results like control	Complete in 10 days fast and partial in 15 days fast	(Braz et al., 2022)
5-day fast and 37 days of refeeding	Nile tilapia (*Oreochromis niloticus*)	− Significant increase in muscle fiber frequency − Expression of muscle growth-related genes	Complete	(Nebo et al., 2013)
1–3 weeks of fasting and 10 weeks of refeeding	Nile tilapia (*Oreochromis niloticus*)	− Reduced body mass of fasted fish, and 10 weeks of refeeding resulted in partial compensatory growth of body mass − igf-1 receptor, ubiquitin ligases *mufr1* and *atrogin-1* expression as well as *myogenin* expression increased during the 1–3 weeks of fasting	Partial	(Nebo et al., 2017)
1 weeks fasting and 5 weeks refeeding	Nile tilapia (*Oreochromis niloticus*)	− Decrease in body weight during fasting − Gene expression levels of *gh*, *mstn*, *myog*, and *npya* were significantly increased, while *igf-1* was markedly depressed in fasted fish − Refeeding restored the above-mentioned genes and body weight	Complete	(Elbialy et al., 2022)
1,2,4 weeks fasting and refeed to 7, 6, 4 respectively	Hybrid tilapia, *Oreochromis mossambicus* × *O. niloticus*	− 1 week had similar body weights to the controls, whereas fish deprived for 2 and 4 weeks had significantly lower body weights than the controls − Feed intake upon refeeding were higher the longer the duration of deprivation − No significant differences were found in digestibility, feed efficiency or protein and energy retention efficiency between the deprived and control fish during refeeding	Complete in 1 week fast and partial in 2 and 4 weeks fast	(Wang et al., 2000)
2 weeks fast and equal refeed	Pejerrey (*Odontesthes bonariensis*)	− Muscle growth mechanisms, hyperplasia and hypertrophy are differentially regulated	Complete	(Simó et al., 2022)
14-day fast, 14 days refeed	Grass carp (*Ctenopharyngodon idella*)	− Fish gained weight continuously during refeeding − The muscle fiber diameter decreased gradually during fasting and then increased with refeeding	Complete	(Xu et al., 2019)
1, 2, 3, or 4-week fast, 20 days refeed (4 weeks)	Persian sturgeon (*Acipenser persicus*)	− Body weight, SGR, K, and HSI significantly decreased	Complete in short fast and partial in long fast	(Yarmohammadi et al., 2013)
8-week fast, 5 weeks refeed	Atlantic salmon (*Salmo salar*)	− 7.3% reduction in body mass; negative SGR; continued length growth; K declined from 1.2 to 1.0	Partial	(Hvas et al., 2022)
30-day fast, 30 days refeed	Hybrid grouper (*Epinephelus fuscoguttatus*♀×*E. lanceolatus*♂)	− Decreased growth performance parameters, weight gain and SGR, while the feeding rate increased during the refeeding	None	(Liu et al., 2020)
45-day fast, 14 days refeed	*Colossoma macropomum*	− Mobilization of body reserves − Declines in final weight and SGR	Partial	(Porto et al., 2024)
101-day fast, 126 days refeed	Arctic charr (*Salvelinus alpinus*)	− Declines in final length, weight, weight gain, SGR and HSI	Partial	(Cassidy et al., 2018)
15 days fasting and 5 days refeeding	lean pacu (*Piaractus mesopotamicus*)	− Higher weight gain and better feed conversion ratio	Complete	(Favero et al., 2018)

Medium-term fasting imposes more substantial effects on growth, typically causing reductions in weight and SGR as fish rely heavily on internal energy reserves to sustain metabolic demands (Hvas et al., 2020). Although this duration induces greater physiological stress than short-term fasting, some species such as Grass carp are still capable of full CG following refeeding (Xu et al., 2019). However, many others—including gilthead seabream and Persian sturgeon—exhibit partial recovery despite the activation of compensatory mechanisms (Yarmohammadi et al., 2013). These responses highlight species-specific resilience and differing capacities for growth restitution under medium-term feed restriction.

In contrast, extended fasting generally impairs growth performance across species, as evidenced by declines in body weight, final length, weight gain, and SGR due to depletion of energy reserves, particularly during early developmental stages, can lead to nutritional deficiencies that impair the organism’s capacity to fully recover even when optimal feeding conditions are reinstated (Metcalfe and Monaghan, 2001). Weight loss in fish tends to be most pronounced during the initial phase of fasting, gradually tapering off in the subsequent weeks. This pattern is attributed to a series of time-dependent physiological and biochemical adaptations that progressively lower metabolic rates (Hvas et al., 2020). Key mechanisms that help conserve energy under fast conditions include alterations in gene expression, reduced muscle enzyme activity, and suppression of protein synthesis (Salin et al., 2018; Zhao et al., 2022; Cheng et al., 2024). Due to these dynamic physiological adjustments, accurately predicting how long fish can endure fasting remains challenging. Although CG may still occur after extended fasting, recovery often requires prolonged refeeding and may not fully restore growth trajectories prior to harvest (Hvas et al., 2022). Consequently, predicting tolerance limits for fasting remains challenging and must be evaluated within a species-specific physiological and production context.

## 3 Factors affecting compensatory growth

The extent of growth loss and the potential for CG in fish are influenced by several factors, including the duration and intensity of fasting, species-specific traits, developmental stage, environmental conditions, and refeeding strategy (Torfi Mozanzadeh et al., 2015; Torfi Mozanzadeh et al., 2021; Braz et al., 2022). Among these, the severity and duration of feed restriction are particularly critical. Short periods of feed deprivation often induce strong compensatory responses upon refeeding, as energy reserves remain sufficient to support rapid metabolic recovery and growth acceleration (Nebo et al., 2013; Oh et al., 2013; Tamadoni et al., 2020). These responses highlight the importance of feeding pattern rather than only fasting duration, as intermittent feeding strategies have been widely investigated as a tool to regulate growth performance in aquaculture. In such regimes, brief and controlled feed deprivation followed by refeeding is generally well tolerated, as metabolic homeostasis is initially maintained through glycogen and lipid mobilization. Upon refeeding, many fish species exhibit compensatory growth characterized by increased feed intake (hyperphagia) and improved feed conversion efficiency, which can enhance growth recovery. However, the overall effectiveness of these feeding cycles depends on their frequency, duration, and species-specific metabolic capacity, as excessive repetition may reduce compensatory potential.

In contrast, prolonged fasting induces more pronounced metabolic stress, including depletion of lipid and glycogen reserves and increased reliance on protein catabolism. As a result, the capacity for full compensatory growth is often reduced, and recovery may be slower or incomplete even under optimal refeeding conditions. These differences highlight that the duration and pattern of feed deprivation are critical determinants of compensatory growth efficiency and should be carefully considered in aquaculture management strategies (Torfi Mozanzadeh et al., 2021; Hvas et al., 2022; Porto et al., 2024). Consequently, while short to moderate fasting periods frequently result in partial or complete compensatory growth, extended deprivation may impair physiological condition and reduce the efficiency of growth recovery.

Species and developmental stage also significantly affect CG capacity. Juvenile fish are often reported to exhibit strong compensatory growth following periods of feed restriction due to their high growth potential and greater physiological plasticity during early life stages. Rapid tissue accretion and flexible energy allocation strategies allow juveniles to redirect nutrients toward somatic growth when feeding resumes (Jobling, 2010). However, this response is not universal and may be constrained by the high metabolic demands’ characteristic of early developmental stages. Juvenile fish generally display elevated metabolic rates compared with adults, which increases their sensitivity to food deprivation and accelerates the depletion of endogenous energy reserves during fasting. For instance, (Wang et al., 2000) found that juvenile Nile tilapia exposed to repeated short fasting cycles (three days each week) experienced physiological stress that limited growth recovery, illustrating that even early life stages do not always exhibit strong compensation. Consequently, prolonged or severe feed restriction may impair the ability of juveniles to fully compensate for growth losses despite their high growth potential (Metcalfe and Monaghan, 2001). The magnitude of compensatory growth in juvenile fish therefore depends on the interaction between growth plasticity, metabolic demand, fasting duration, and species-specific physiological traits. These factors contribute to the variability reported across studies, where juveniles under certain conditions exhibit full compensatory growth, while in other cases only partial or no compensation is observed (Nicieza and Metcalfe, 1997).

Dietary restriction during early developmental stages can have lasting effects on subsequent muscle growth and overall growth performance in fish. Experimental evidence indicates that early feed restriction may reduce initial muscle fiber recruitment (hyperplasia) and slow growth; however, subsequent refeeding can stimulate compensatory mechanisms, including increased muscle fiber size (hypertrophy) and partial recovery of growth. For example, studies in teleost fish have shown that restricted feeding during juvenile stages alters muscle growth dynamics, affecting both fiber number and diameter during later growth phases (Rescan et al., 2007; Valente et al., 2013). Nevertheless, the long-term outcome depends on the severity and duration of the restriction. Moderate early-life restriction followed by adequate refeeding may allow substantial growth compensation, whereas severe or prolonged restriction can impair muscle development and reduce growth potential at later stages. These findings highlight that early nutritional history plays a critical role in shaping muscle growth trajectories and influences the efficiency of compensatory growth responses in fish.

Environmental factors play a crucial role in shaping the potential for growth recovery in fish following fasting (Oh et al., 2013; Braz et al., 2022). Environmental temperature represents an important factor influencing compensatory growth responses in fish because it strongly regulates metabolic rate and energy expenditure. As ectothermic organisms, fish exhibit temperature-dependent metabolic rates, which directly affect energy utilization and digestive processes. Experimental studies have shown that low temperatures can slow gastrointestinal evacuation during fasting and are associated with reduced metabolic activity and nutrient turnover, reflecting a metabolic down-regulation that helps conserve energy reserves during food deprivation (Waagbø et al., 2017). In addition, temperature can modify the magnitude and timing of metabolic adjustments during starvation. Comparative studies on *Lota lota* and *Rutilus rutilus* demonstrated that starvation can induce a marked depression in metabolic rate under warm conditions, whereas similar reductions were not observed at lower temperatures. Moreover, energy expenditure during fasting tended to be highest at temperatures corresponding to the natural active periods of each species, indicating that thermal adaptation influences how fish regulate energy turnover during food deprivation. These responses may also involve behavioral adjustments, such as reduced activity, and differential reliance on energy reserves from muscle or liver tissues (Binner et al., 2008). However, interpreting the role of temperature requires caution, as it often interacts with other experimental variables such as fasting duration and feeding intensity during refeeding. For example, in the study by (Braz et al., 2022) all experimental groups were maintained under the same low-temperature conditions, yet reduced compensatory growth was observed only in fish subjected to prolonged feed restriction. This suggests that the duration of fasting, rather than temperature alone, may have contributed significantly to the observed response. Consequently, compensatory growth outcomes likely reflect the combined effects of environmental conditions, species-specific thermal adaptations, and feeding regime characteristics rather than a single environmental factor.

Diet quality and feeding strategy during recovery also play central roles. High-quality diets combined with ad libitum feeding significantly enhance feed utilization and promote accelerated growth (Eroldoğan et al., 2006; Yılmaz and Eroldogan, 2011). These variables underscore the multifaceted nature of CG and emphasize the importance of tailoring feeding protocols to specific species and conditions in aquaculture to optimize recovery and performance.

## 4 Physiological and molecular mechanisms of compensatory growth

The CG response in fish is governed by a multifaceted interaction of physiological, metabolic, and endocrine mechanisms. A key contributor to this response is hyperphagia which facilitates enhanced nutrient assimilation and supports accelerated somatic growth (Rueda et al., 1998; Wang et al., 2006; Oh and Park, 2019; Kim et al., 2022). This phase is also marked by increased activity of digestive enzymes in some species, further optimizing nutrient breakdown and absorption (Caruso et al., 2014; Zhao et al., 2024). Additionally, fish undergoing CG often demonstrate improved FCE, enabling a greater proportion of ingested energy to be allocated toward tissue accretion rather than maintenance metabolism (Kim et al., 2022).

The synergistic interaction between elevated feed intake and improved nutrient utilization efficiency during refeeding enables certain fish species to exhibit partial or complete CG. However, the magnitude, duration, and efficiency of feed conversion recovery are markedly species-specific and context-dependent. For instance, Juvenile Nile tilapia (*Oreochromis niloticus*) and leopard mandarin fish (*Siniperca scherzeri*) exhibited full CG recovery accompanied by significant modulation of muscle growth-related gene expression after brief fasting–refeeding cycles (Nebo et al., 2013). Similarly, juvenile leopard mandarin fish (*Siniperca scherzeri*) demonstrated complete catch-up growth after fasting (Kim et al., 2022). Marine species such as blackhead seabream (*Acanthopagrus schlegelii*) also achieved full CG under restricted feeding strategies in sea cages (Oh et al., 2013). In contrast, European sea bass (*Dicentrarchus labrax*) showed incomplete growth recovery after repeated fasting–refeeding cycles, with responses influenced by the duration and frequency of restriction (Adaklı and Taşbozan, 2015). Furthermore, variable compensatory patterns have been reported in Persian sturgeon (*Acipenser persicus*), where short fasting periods stimulated growth recovery, whereas prolonged deprivation reduced the efficiency of compensation and altered *igf-1* gene expression (Yarmohammadi et al., 2013). Collectively, these findings clearly demonstrate that CG is not a uniform biological response across fish species. Instead, it is strongly influenced by species-specific metabolic capacity, digestive plasticity, endocrine regulation, and the duration and intensity of fasting protocols. Therefore, fasting–refeeding strategies must be tailored to individual species and production contexts rather than generalized across aquaculture systems.

At the metabolic level, fasting initiates a coordinated shift in energy metabolism aimed at maintaining metabolic homeostasis and glucose balance. Fasting induces the mobilization of energy reserves, including glycogen, lipids, and, to a lesser extent, proteins. As glycogen stores become depleted, gluconeogenesis becomes a critical pathway for endogenous glucose production, primarily in the liver. This process utilizes non-carbohydrate substrates such as amino acids (e.g., alanine and glutamine), glycerol derived from lipid mobilization, and lactate (Favero et al., 2018; He et al., 2022). The relative contribution of each substrate differs across taxa and feeding strategies. For instance, studies on Nile tilapia suggest that lipids and hepatic glycogen may constitute major energy sources during fasting (Hsieh and Shiau, 2000). Notably, tilapia exhibit a high degree of physiological adaptability to extended periods of feed restriction and subsequent refeeding, making them particularly resilient under fluctuating nutritional conditions. However, in many carnivorous fish species, carbohydrate metabolism is limited, and energy demands during fasting are largely met through lipid mobilization and, to a lesser extent, protein catabolism (Cleveland and Radler, 2019; Francesca et al., 2020). Consequently, the sequence and magnitude of energy reserve utilization depend strongly on species-specific metabolic strategies.

In some fish species, prolonged fasting is associated with a glycogen-sparing effect, whereby lipid oxidation is preferentially enhanced to conserve limited carbohydrate reserves. This metabolic strategy reduces reliance on glycogen stores and delays the onset of protein catabolism, thereby contributing to improved survival during extended periods of food deprivation. However, the extent of this response varies among species depending on their metabolic flexibility and ecological adaptation (Hoseini et al., 2019).

Fasting and refeeding also significantly affect lipid metabolism, including circulating cholesterol and triglyceride levels. Cholesterol, a key structural lipid, plays an essential role in steroid hormone synthesis such as cortisol, which regulates energy metabolism and stress responses (Mommsen et al., 1999). Its levels during fasting may vary depending on species-specific metabolic strategies, with reports showing decreases in some fish species (Kim et al., 2020; Kim et al., 2021; Kim et al., 2022; Messina et al., 2023), stability in others (Silva et al., 2019; Assis et al., 2020; Favero et al., 2021), or even increases in certain species such as climbing perch and pacu (Godavarthy et al., 2012; Favero et al., 2018). These differences reflect variations in metabolic demands, stress responses, and ecological adaptations.

During fasting, triglycerides serve as the primary lipid energy reserve and are mobilized through enhanced lipolytic activity to support energy production. The breakdown of triglycerides releases glycerol, which contributes to gluconeogenesis, while free fatty acids undergo β-oxidation to generate ATP (Grigorakis and Alexis, 2005; Schweiger et al., 2006; Morash and McClelland, 2011). Consequently, many studies report a decline in circulating triglyceride levels during fasting, reflecting increased lipid utilization (Liu et al., 2022; Messina et al., 2023). However, opposite patterns have also been observed in some species, where elevated triglyceride levels may indicate enhanced lipid mobilization under energetic stress (Norouzi et al., 2020). Overall, lipid responses during fasting are highly species-dependent and closely linked to environmental conditions and metabolic flexibility.

Fasting is associated with dynamic changes in circulating amino acid profiles, reflecting the mobilization of endogenous protein reserves. Although protein catabolism is often considered a secondary response after carbohydrate and lipid utilization, evidence indicates that glycogenolysis, lipolysis, and proteolysis may occur concurrently depending on species-specific metabolic strategies (Mommsen et al., 1999; McCue, 2010). During prolonged fasting, enhanced proteolysis contributes to amino acids as substrates for energy production and gluconeogenesis. This process is mediated by increased activity of proteolytic systems such as the ubiquitin–proteasome pathway and cathepsins, alongside elevated amino acid–catabolizing enzymes including alanine aminotransferase (ALT) and aspartate aminotransferase (AST) (Peragón et al., 1999; Yarmohammadi et al., 2015).

Consequently, circulating amino acid levels may fluctuate during fasting depending on the balance between protein breakdown and utilization for metabolic needs. Species with limited lipid reserves tend to rely earlier on amino acid catabolism, whereas lipid-rich species exhibit delayed protein mobilization (Furné et al., 2012). Upon refeeding, amino acid profiles are restored as dietary protein intake resumes and anabolic processes are reactivated, supporting protein synthesis and tissue recovery.

Morphological indices also reflect metabolic adjustments during fasting. (Messina et al., 2023) documented reductions in viscerosomatic (VSI) and hepatosomatic indices (HSI) during feed deprivation, consistent with depletion of liver reserves. Upon refeeding, a rapid increase in body weight is typically observed in the initial days, largely attributed to gut filling. This is followed by partial CG, characterized by the regeneration of digestive tissues and replenishment of hepatic glycogen, although the extent of these processes differs among species.

However, this initial increase in body weight is transient and does not fully reflect true tissue accretion. As refeeding progresses, weight gain becomes increasingly associated with nutrient deposition, including both lipid accumulation and muscle growth. Experimental studies have shown that fasting induces reductions in muscle fiber size, followed by increased muscle fiber diameter and hypertrophy during refeeding, indicating active muscle growth recovery (Xu et al., 2019; Khormi et al., 2026). In contrast, other studies have reported increased lipid accumulation and reduced muscle protein content following fasting–refeeding regimes, suggesting that compensatory growth may involve preferential fat deposition depending on species and feeding conditions (Xiong et al., 2026). Consequently, fasting–refeeding strategies may alter carcass composition and affect flesh quality. Changes in muscle structure and metabolic profiles during compensatory growth have been associated with variations in fillet characteristics and overall meat quality (Gabriel Kuniyoshi et al., 2019).

Refeeding generally shifts metabolism toward anabolic processes that support tissue growth and repair (Cassidy et al., 2018), mediated in part by endocrine signals such as growth hormone, insulin-like growth factors (Igfs), and thyroid hormones (Davis and Gaylord, 2011). During this phase, increased feed intake and improved nutrient assimilation can stimulate protein synthesis and somatic growth. In some species, fasting has also been associated with reductions in maintenance metabolism, which may improve growth efficiency once feeding resumes (Hvas et al., 2020). However, the magnitude and pattern of compensatory growth responses are highly variable and cannot always be attributed solely to intrinsic species-specific traits. In many experimental studies, differences in compensatory responses are closely linked to feeding strategy–dependent factors, including fasting duration, feeding intensity during refeeding, developmental stage, and environmental conditions. These factors influence the balance between endocrine signaling, metabolic regulation, and nutrient availability, thereby determining whether fish exhibit full, partial, or absent compensatory growth. Consequently, understanding compensatory growth requires an integrative evaluation of both physiological regulation and experimental design variables rather than attributing variability exclusively to species differences.

At the molecular level fasting and refeeding induce marked gene expression changes that directly influence muscle metabolism and growth in fish (Nebo et al., 2013). Nutrient availability is a key regulator of skeletal muscle development, mainly through modulation of the GH/IGF-1 axis (Liu et al., 2020), which controls nutrient utilization and protein synthesis (Butler and Le Roith, 2001). In several species, fasting has been associated with elevated growth hormone levels and reduced hepatic and muscular *igf-1* expression, a pattern often linked to reduced anabolic signaling and increased catabolism. However, the magnitude and direction of these responses differ among species, tissues, and developmental stages (Waagbø et al., 2017; Chen et al., 2019). This disruption of the GH/IGF-1 axis suppresses muscle cell proliferation and promotes catabolic processes. Reduced *igf-1* has been associated in some studies with enhanced expression of muscle-specific ubiquitin ligases such as *murf1* and *atrogin-1*, contributing to muscle atrophy and growth impairment during prolonged feed deprivation (Nebo et al., 2017). Following refeeding, restoration of *igf-1* expression may contribute to renewed anabolic signaling. The *igf-1* has been shown in fish, as in other vertebrates, to activate PI3K/Akt-related pathways associated with protein synthesis and muscle hypertrophy (De-Santis and Jerry, 2007; Rescan et al., 2007). Nevertheless, CG is likely regulated by multiple interacting pathways rather than a single endocrine signal.

Several genes are implicated in the regulation of skeletal muscle growth in fish, particularly *myostatin* (*mstn*) and members of the myogenic regulatory factor (MRF) family. Myostatin, a member of the transforming growth factor-β (TGF-β) superfamily, is widely recognized as a negative regulator of muscle development and has been associated with reduced muscle growth and muscle atrophy in both mammals and fish (de Santis et al., 2012; Nebo et al., 2013). In contrast, the myogenic regulatory factors, including *myod*, *myf5*, *myogenin* (*myog*), and *mrf4*/*myf6* play central roles in the control of myogenesis by regulating the determination, proliferation, and differentiation of muscle precursor cells through activation of muscle-specific gene transcription. Homologues of these transcription factors have been identified in several lower vertebrates, including teleost fish, where they form part of a complex regulatory network controlling skeletal muscle formation and development (Rescan, 2001). Experimental studies in teleosts have further demonstrated that the expression of muscle-related genes is strongly influenced by nutritional status. During periods of feed deprivation, growth is suppressed and skeletal muscle undergoes metabolic and molecular adjustments associated with reduced anabolic activity. However, the transition from fasting to refeeding can stimulate the expression of several genes involved in muscle growth and cellular differentiation, indicating activation of pathways associated with myogenesis and protein synthesis (Bower and Johnston, 2010). Similarly, studies on rainbow trout have shown that starvation and subsequent refeeding alter the expression of both metabolic-related and muscle-specific genes, including MRFs, myostatins, and structural proteins such as myosin. These transcriptional changes are thought to promote muscle remodeling and hypertrophic growth of myotubes, thereby contributing to the accelerated growth frequently observed after refeeding (Johansen and Overturf, 2006). Such nutritionally regulated gene expression patterns provide insight into the molecular mechanisms through which fasting and subsequent refeeding modulate skeletal muscle growth in fish.

Myogenic regulatory factors including *myod*, *myf5*, *myogenin*, and *mrf4/myf6* play central roles in the regulation of myogenesis in teleost fish through coordinated control of myoblast proliferation and differentiation. Among these factors, myogenin is primarily associated with the differentiation stage of muscle development, whereas MyoD and Myf5 are mainly involved in the determination and proliferation of myogenic precursor cells. Several studies in teleosts have shown that the expression of these regulatory factors is modulated during muscle growth and remodeling, particularly under nutritional manipulations such as fasting and refeeding (Mommsen et al., 1999; Kim et al., 2020; Kim et al., 2021).

Fasting has been shown to upregulate *mstn* and *myod* expression, while refeeding reverses this effect in tilapia (Elbialy et al., 2022), suggesting a compensatory activation of myogenic pathways. Myostatin is known to inhibit differentiation by suppressing myogenin, making MRFs likely downstream targets of *mstn* (Joulia et al., 2003). This relationship is not universal; in rainbow trout, both myogenin and *mstn* decreased after prolonged fasting and increased upon refeeding, indicating that *mstn* may not regulate myogenin (Johansen and Overturf, 2006). The mechanistic role of *mstn* in fish muscle growth remains incompletely understood and appears to depend on factors such as species, developmental stage, muscle type, and nutritional status (Patruno et al., 2008).

Several central neuroendocrine pathways regulate food intake and energy metabolism, with the hypothalamus integrating peripheral and central signals to modulate appetite. Among them, neuropeptide Y (Npya) is a key orexigenic factor that promotes feeding and energy homeostasis in teleosts (Soengas et al., 2018). Increased hypothalamic *npy* expression during fasting has been documented in several species and refeeding subsequently normalizes its expression (Ji et al., 2015; Rescan et al., 2017), supporting its role in stimulating appetite post-fasting for CG, although patterns vary across taxa.

Leptin also plays an important role in energy regulation during starvation and is linked to metabolism, growth, and appetite control (Denver et al., 2011). Its response to fasting is inconsistent among fish species. *leptin* expression reduced in some taxa during feed deprivation (Yuan et al., 2016; Dar et al., 2018), suggesting a mechanism to reduce metabolic demand under nutrient deprivation. Upon refeeding, *leptin* expression increases, reflecting improved nutritional status and metabolic demand, although levels may decline after prolonged recovery (Sakyi et al., 2020). Other studies show stable or increased levels, suggesting that *leptin* function in fish differs from the classical mammalian model (Tinoco et al., 2012; Wu et al., 2025).

## 5 Impacts of fasting strategies on aquacultures

In aquaculture, the biological response to feeding is crucial. Recognizing the role of CG is pivotal for advancing aquaculture practices. Evidence from multiple studies indicates that carefully controlled fasting periods may, in some circumstances, allow fish to recover growth after refeeding without major reductions in final size. However, outcomes depend strongly on species, life stage, environmental conditions, and feeding management during recovery (Elbialy et al., 2022; Liu et al., 2022). Strategically timed fasting can enhance feed management by reducing waste, lowering water pollution, and promoting animal welfare (Braz et al., 2022; Peng et al., 2026). In addition, compensatory responses may contribute to improved production efficiency by promoting partial convergence in growth rates among individuals, which can reduce size variability within cultured populations and facilitate more uniform harvest sizes (Tamadoni et al., 2020; Simó et al., 2022; Refaey et al., 2024). Consequently, such responses may also contribute to lowering production costs while maintaining acceptable growth performance.

The observation that fasted fish may exhibit CG prior to harvest suggests that continuous daily ad libitum feeding may not be the only strategy capable of supporting acceptable production outcomes. Accelerated growth following feed restriction highlights the regulated nature of somatic growth and may contribute to reduced size variability through partial convergence among individuals over time (Simó et al., 2022; Refaey et al., 2024). Nevertheless, the present findings should not be interpreted as a direct comparative evaluation of feeding regimes. Comprehensive assessments incorporating SGR, feed conversion ratio, protein efficiency ratio, survival, health status, and economic return under controlled comparisons are required before making practical recommendations. Under carefully managed conditions, intermittent feed omission (e.g., short non-feeding intervals) may not necessarily compromise final harvest size; however, this remains dependent on species, culture conditions, and management practices.

## 6 Conclusion

This review synthesizes current knowledge on fasting and refeeding responses in fish and highlights the complex physiological, metabolic, and molecular mechanisms underlying compensatory growth (CG). Fasting induces coordinated metabolic adjustments characterized by energy conservation and mobilization of lipid and protein reserves, whereas refeeding activates anabolic pathways, growth-promoting genes, and endocrine regulators that facilitate growth recovery. Hyperphagia and enhanced feed efficiency are central drivers of this response. However, CG is highly species-specific and influenced by fasting duration, developmental stage, environmental conditions, and post-refeeding feeding intensity. While short to moderate fasting periods may trigger beneficial compensatory responses, prolonged deprivation can impair physiological integrity and limit full growth recovery. These interspecific differences underscore the importance of tailored feeding strategies rather than generalized protocols. From an applied aquaculture perspective, understanding the variability of compensatory growth responses is essential for designing feeding strategies that optimize production efficiency. Controlled comparisons of feeding regimes can help identify management practices that balance feed input, growth performance, and production costs under different farming conditions. Such knowledge may assist producers in implementing feeding schedules that maintain growth while potentially improving feed conversion efficiency. Despite the progress achieved, several research gaps remain. In particular, further studies are needed to clarify the relative contribution of environmental factors, fasting duration, and feeding strategies in determining compensatory growth outcomes across species. In addition, deeper molecular investigations of endocrine and metabolic regulatory networks are required to better understand the physiological limits of compensatory growth. Addressing these knowledge gaps will help refine feeding strategies and support the development of more sustainable aquaculture practices.
